# Hydrocele Cured by Electricity

**Published:** 1846-02

**Authors:** 


*Hydrocele Cured by Electricity.*—Dr. F. Campbell Stewart mentioned having recently cured a large hydrocele, seventeen inches in circumference, and thirteen and a half from pubis to its base, by *electricity;* one needle was inserted into the cavity of the tunica vaginalis testis, also one through the cellular tissue over the scrotum. The application of electricity was continued half an hour, and was attended with intense pain. In forty-eight hours the fluid had entirely disappeared. A second case treated in a similar manner was not successful.—*Proceedings of the N. Y. Med. and Surg. Society.*

